# A Rare Case of Hormone-Induced Plasma Cell Granuloma of the Gingiva

**DOI:** 10.7759/cureus.23897

**Published:** 2022-04-06

**Authors:** Dyna Albert, Muthusekhar M.R., Santhosh P Kumar, Murugesan Krishnan

**Affiliations:** 1 Oral and Maxillofacial Surgery, Saveetha Dental College and Hospital, Chennai, IND

**Keywords:** gingival growths, gingival neoplasms, benign tumor, hormone therapy, plasma cell granuloma, plasma cell tumor

## Abstract

Plasma cell granuloma (PCG), also known as inflammatory pseudotumor, is of unknown etiopathogenesis. It commonly presents in the lungs and can also occur in the liver, kidney, brain, and heart. PCG is rare in the oral cavity and even rarer in the gingiva. The clinical and radiological presentation of this disease in the oral cavity appears to be aggressive in nature and is often misdiagnosed as a malignant lesion. Histopathology helps in distinguishing PCG of gingiva from other benign and malignant lesions of the gingiva. Amlodipine and cyclosporine-induced PCG of gingiva have been reported in the literature. This report presents a rare case of generalized plasma cell granuloma of the gingiva in an adult female patient who was on hormonal therapy for infertility. Treatment consisted of complete surgical excision of the lesion and extraction of teeth with a poor prognosis. Wound healing was uneventful during the one-year follow-up period with no signs of recurrence.

## Introduction

Plasma cell granuloma (PCG) is also known as inflammatory pseudotumor and is of unknown etiopathogenesis. It commonly presents in the lungs and can also occur in the liver, kidney, brain, and heart [1]. In the head and neck region, it can occur in the orbit, tonsils, paranasal sinus, and the thyroid gland. PCG is rare in the oral cavity and rarer in the gingiva [2]. The lesion is formed by the accumulation of polyclonal plasma cells in a background of fibrosis and spindle cell proliferation [1]. PCG has no predilection for age or gender with equal incidences for maxillary and mandibular gingivae. It usually manifests as an asymptomatic, exophytic, solitary, well-demarcated lesion with a tendency to bleed on provocation. Although PCG of gingiva is benign in nature, the clinical and radiological presentation in the oral cavity can mimic malignancy [3]. Although drug-induced PCG of gingiva has been reported in the literature [4,5], there are no reports of PCG of gingiva occurring as a result of hormonal therapy. This report presents a rare case of generalized plasma cell granuloma of the gingiva in a 35-year-old female patient who was on hormonal therapy for infertility.

## Case presentation

A 35-year-old female reported to the Department of Oral and Maxillofacial Surgery with a chief complaint of growth in the upper and lower jaw for the past four years. The patient also complained of teeth mobility in the lower right posterior and upper left posterior teeth region for the past year. History of presenting illness revealed that the growth was initially small and it gradually increased to the present size. The growth was painless and bleeding upon brushing was present. Past medical history revealed that the patient was under hormone therapy for infertility seven years ago for a duration of three years and later was not under any medication. Past dental history revealed that the patient consulted a dental practitioner for the same complaint three years ago and underwent scaling and curettage of the upper and lower teeth.

**Figure 1:**
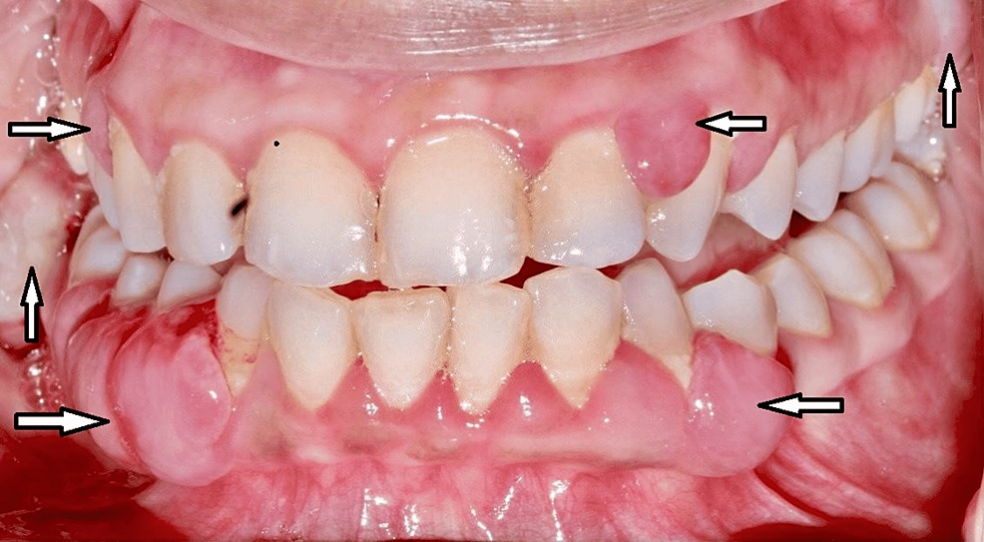
Intraoral view showing gingival lesions in all four quadrants (arrow)

**Figure 2:**
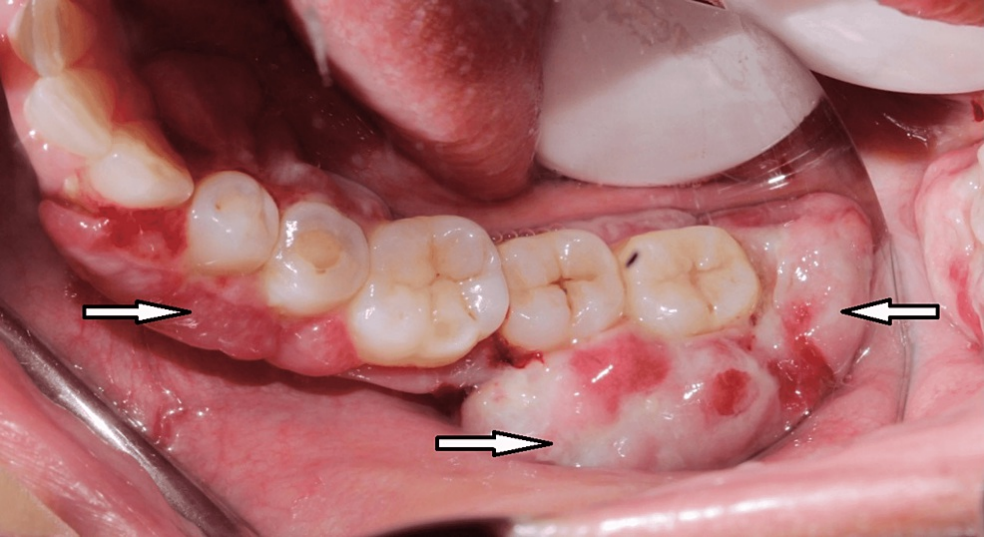
Intraoral view in mirror image showing gingival lesions (arrow) in the fourth quadrant and the teeth associated with the lesions

**Figure 3:**
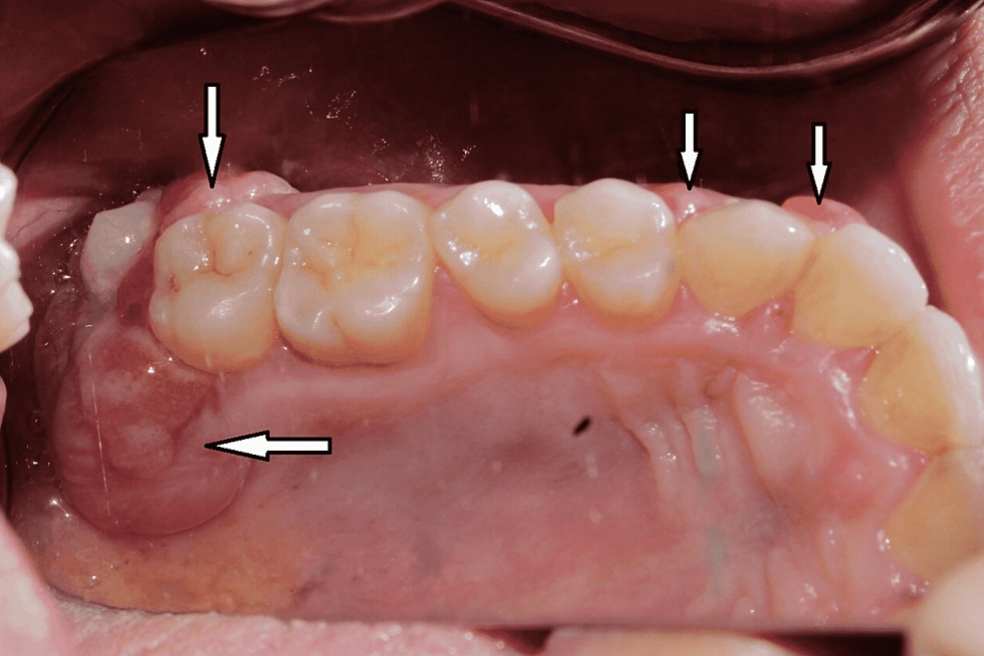
Intraoral view in mirror image showing gingival lesions (arrow) in the second quadrant and the teeth associated with the lesions

Inspection of the lesion showed multiple localized lobulated gingival growth, reddish-pink in color in relation to tooth numbers 43, 44, 45, 47, 48, 13, 14, 23, 24, 27, 33, and 34 (Figure 1). Upon palpation, the lesion was non-tender, edematous, and soft in consistency with bleeding on provocation. Dental examination revealed root canal treated 45 and Class I caries in relation to 16 and 37 and oral hygiene was fair. The periodontal probing depth ranged from 5 mm to 9 mm in relation to 45, 46, 47, 48, 27, and 28. Periodontal examination revealed that 48 (Figure 2) and 28 (Figure 3) were the most affected teeth with grade III mobility followed by 46, 47, and 27 with grade II mobility and 45 with grade I mobility.

**Figure 4:**
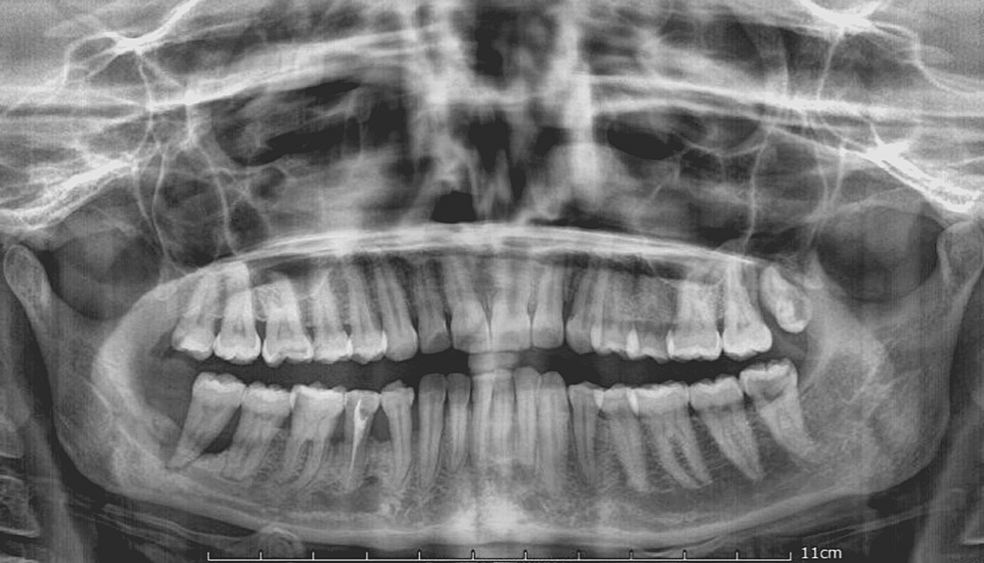
Orthopantomogram showing generalized alveolar bone loss

**Figure 5:**
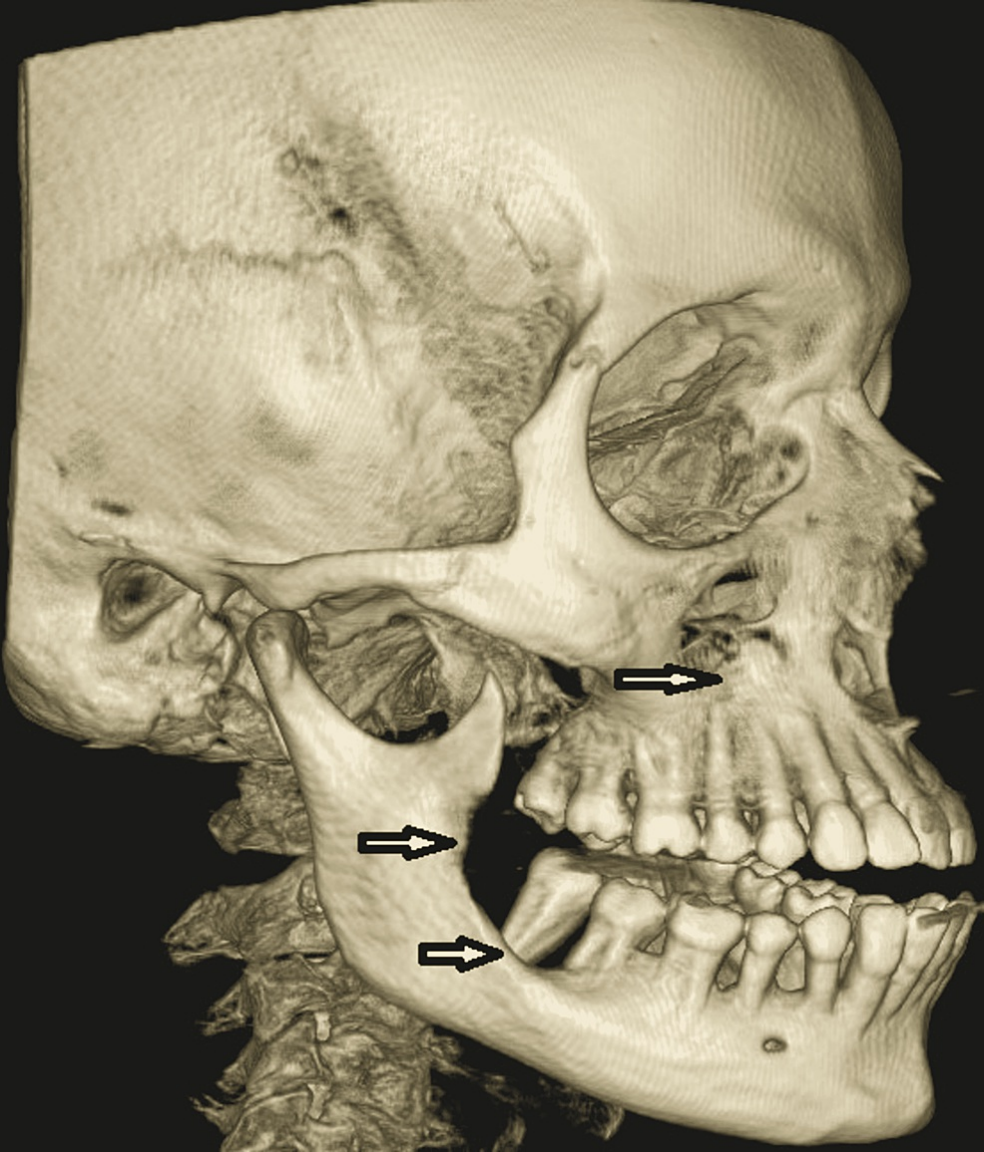
CBCT Scan showing bony erosion in the floor of the right maxillary sinus and ascending ramus of the right mandible (arrow)

Orthopantomogram revealed generalized alveolar bone loss (Figure 4). Cone-beam computed tomography (CBCT) scan of upper and lower jaw revealed osteolytic lesion involving the posterior palate with the erosion of the floor of the right maxillary sinus, ascending ramus of the right mandible, and complete loss of alveolar bone in relation to 28 and 48 which was suggestive of inflammatory, granulomatous or malignant lesion (Figure 5). Blood investigation was done to rule out leukemia which revealed elevated alkaline phosphatase levels of 199 international units per liter (IU/L), indicating bone resorption and periodontitis. A provisional diagnosis of peripheral giant cell granuloma of the upper and lower jaw was made. Incisional biopsy was carried out in relation to 47 and 48 which was suggestive of a plasma cell-associated granuloma. Excision of the lesion with diathermy was planned under general anesthesia.

**Figure 6:**
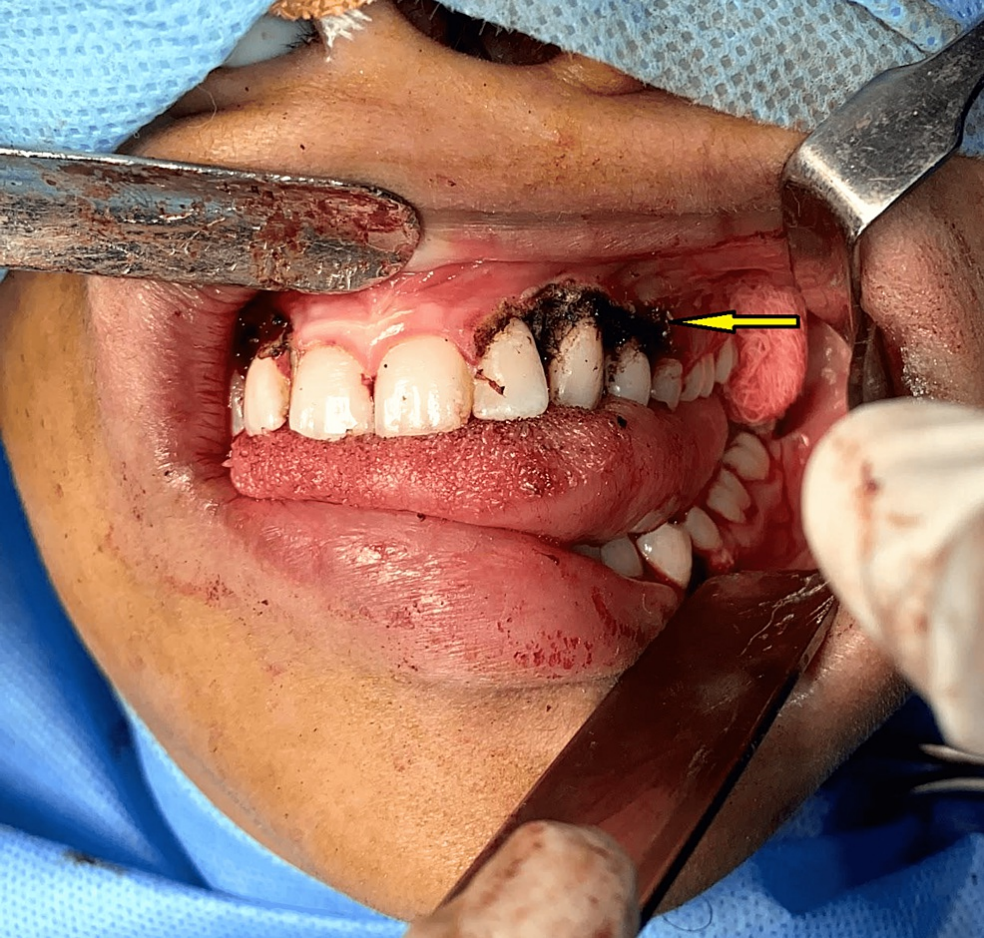
Surgical site after excision of the gingival lesions from the second quadrant (arrow)

**Figure 7:**
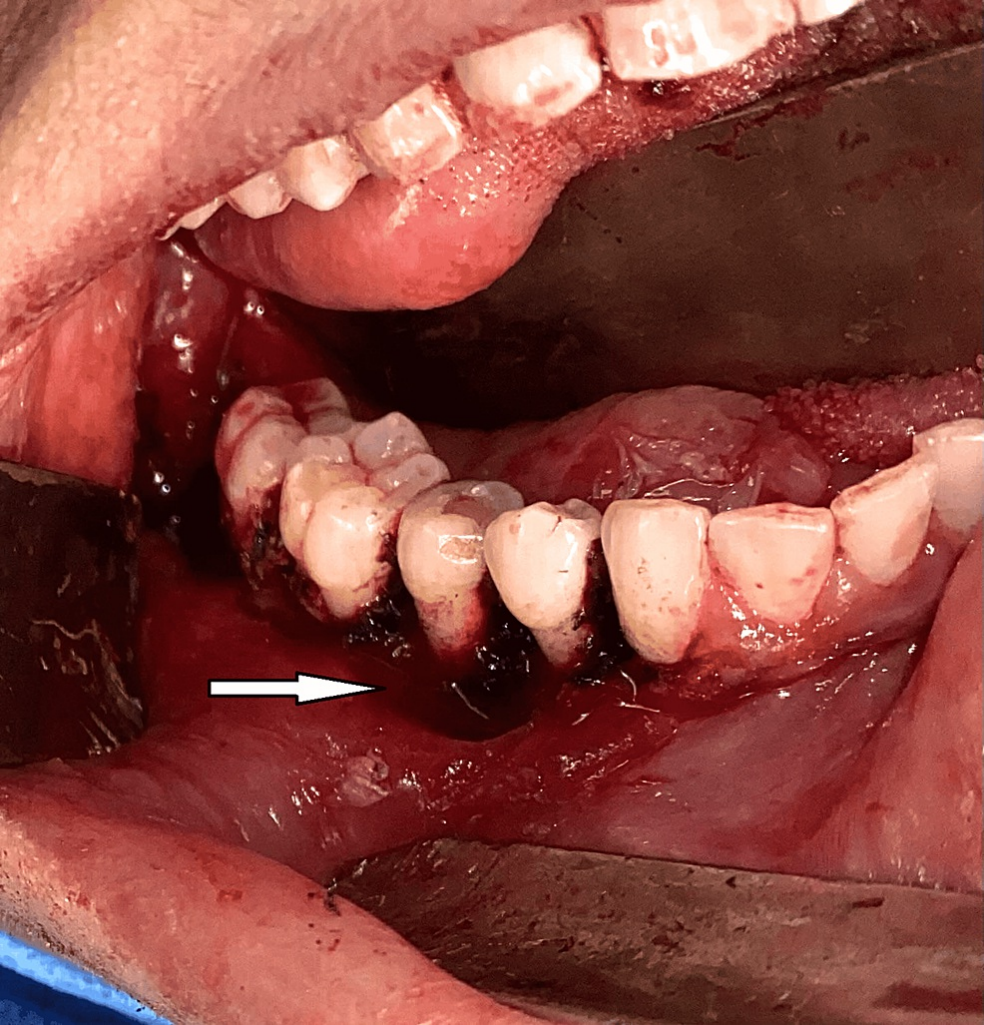
Surgical site after excision of the gingival lesions from the fourth quadrant (arrow)

**Figure 8:**
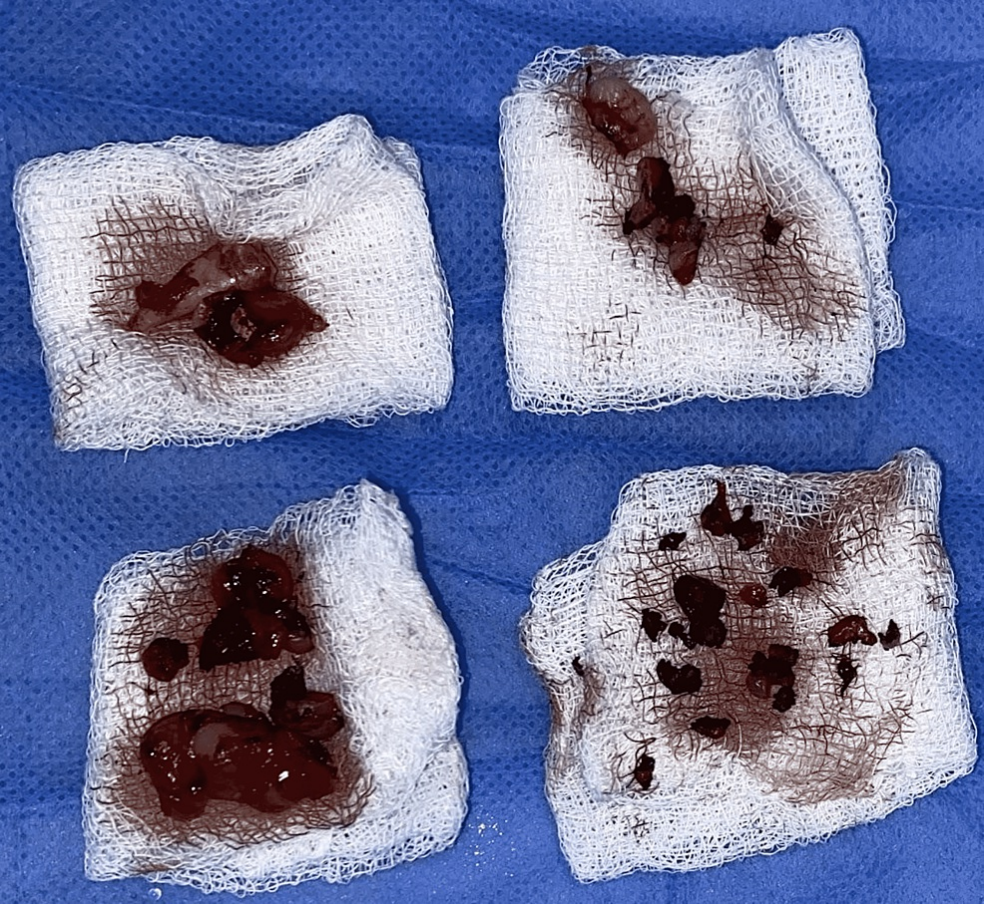
Excised gingival lesions and the extracted teeth

Under general anesthesia, diathermy was used for excision of the lesion from its base in all the quadrants, and hemostasis was achieved (Figures 6, 7). Extraction of teeth 48 and 28 was done. Excised specimens were sent for histopathological examination (Figure 8) which showed mature connective tissue stroma with intense mixed inflammatory cell infiltrate of predominantly plasma cells arranged as sheets and the presence of hyalinization in several areas. Blood vessels of varying size and endothelial proliferation were also noted. The overlying epithelium was para-keratinized stratified squamous epithelium of variable thickness.

**Figure 9:**
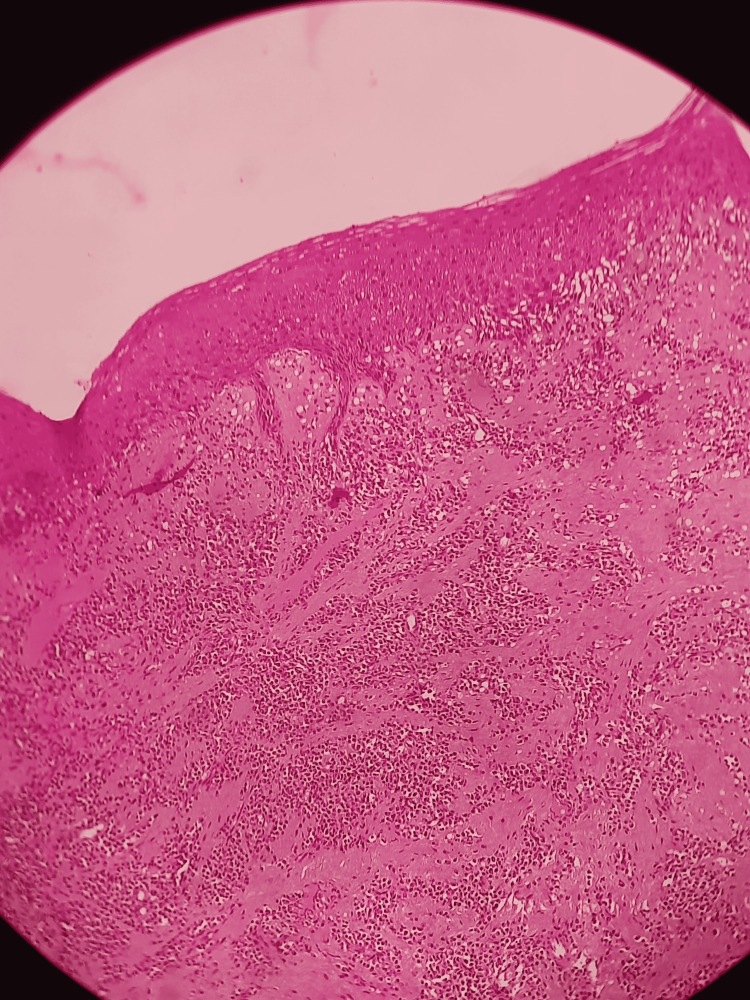
Histological examination showing features typical of plasma cell granuloma of the gingiva (hematoxylin and eosin stain, 100x magnification)

**Figure 10:**
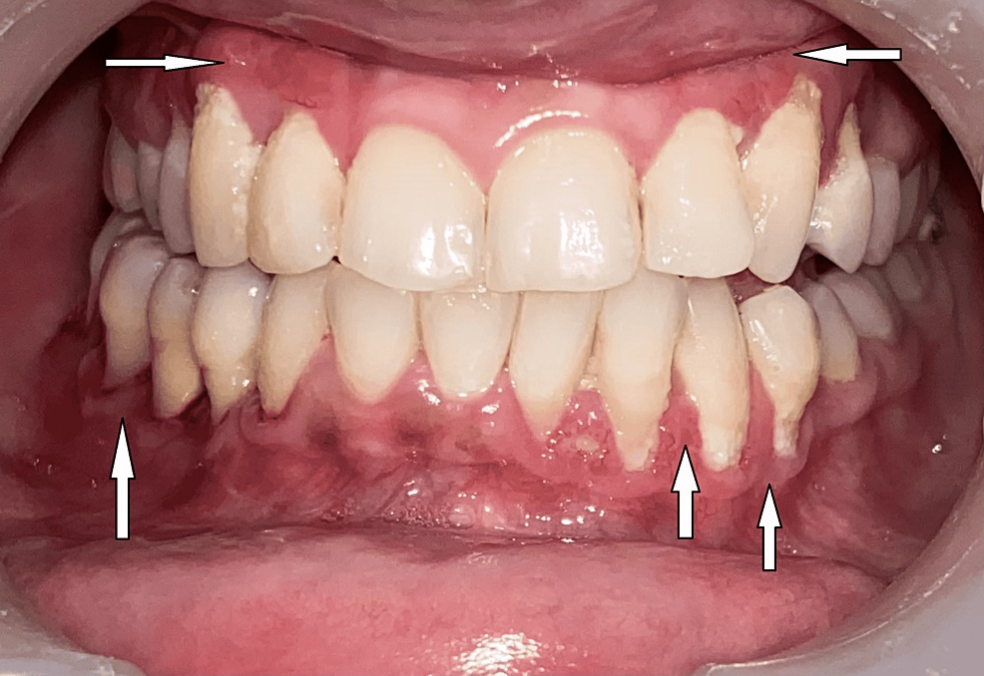
One-month postoperative intraoral examination showing good wound healing (arrow)

Histological examination confirmed the diagnosis of plasma cell-associated granuloma of the gingiva (Figure 9). One-month postoperative review of the patient showed satisfactory wound healing (Figure 10). The patient is under periodic review for one year exhibiting uneventful healing and no signs of recurrence.

## Discussion

Plasma cell tumors include multiple myeloma, solitary myeloma, soft tissue myeloma (plasmacytoma), and plasma cell granuloma. Of the plasma cell tumors, multiple myeloma and solitary myeloma have their origin in the bone while plasmacytoma and PCG originate in soft tissues [6,7]. PCG is also designated as inflammatory pseudotumor, xanthomatous pseudotumor, inflammatory myofibroblastic tumor, and inflammatory myofibrohistiocytic proliferation. PCGs are rare lesions and are an entity of IgG4-related diseases [8]. There is insufficient and unclear evidence regarding its biological behavior and etiopathogenesis. PCG is believed to be inflammatory or autoimmune in origin [9].

Soft tissue plasma cell tumors occur in the oral cavity and respiratory passages. Plasma cell tumors of the gums, lips, buccal mucosa, and tongue are described as atypical gingivostomatitis, allergic gingivostomatitis, and idiopathic gingivostomatitis [10]. Although rigorous allergy testing had been indecisive, the lesions were assumed to be the consequence of a reaction to dentifrices, chewing gum, and other foreign substances [11]. Drug-induced gingival overgrowth is not uncommon and drugs like antiepileptics, calcium channel blockers, and immunosuppressants are known to cause gingival hyperplasia [12]. The influence of hormones like progesterone and estrogen on gingival enlargement is well documented and the increased circulating levels of these hormones in blood during puberty and pregnancy can have adverse reactions to the gingiva [13]. Amlodipine and cyclosporine-induced PCG of gingiva have been reported in the literature [4,5]. However, there is scarcely any report of the occurrence of PCG of gingiva as a result of hormonal therapy in the medical literature. Our patient underwent hormonal therapy with estrogen for a duration of three years for infertility management, resulting in the very rare occurrence of PCG of the gingiva.

PCG of gingiva is very rare and has no predilection for age or gender with equal incidences for maxillary and mandibular gingivae. It usually manifests as an asymptomatic, exophytic, solitary, well-demarcated lesion with a tendency to bleed on provocation [10]. Our patient had a generalized presentation with multiple lesions in all quadrants of the maxilla and mandible. PCG of the oral cavity usually presents with disruption of adjacent tissues, whereas PCG arising in other organs is an incidental finding in radiographs [14]. The ability of PCG for bony infiltration and erosion in the oral cavity mimics malignancy and can often be misleading in diagnosis [15]. Our patient exhibited extensive erosion of bone in the maxilla and mandible in the areas of the lesion.

Unlike PCG which consists of normal plasma cells, plasmacytoma comprises both typical and atypical plasma cells. It is vital to differentiate between plasmacytoma and PCG as the latter is benign while the former may behave more aggressively and progress to multiple myeloma. PCG of the gingiva may mimic conditions like peripheral giant cell granuloma, epulis, pyogenic granuloma, and fibroma [16]. Histopathology provides a confirmative diagnosis for PCG of gingiva and differentiates it from the other plasma cell tumors. Treatment consists of scaling and curettage, complete excision of the lesion, and extraction of teeth with poor prognosis which are present adjacent to the lesion [8]. The prognosis for PCG of gingiva is good due to the low recurrence rate and a long-term follow-up is essential.

## Conclusions

PCG, a rare non-neoplastic lesion of unknown etiology, primarily occurs in the lungs and also has a rarer incidence in the oral cavity. PCG of gingiva is a very rare benign tumor of the oral cavity which may mimic other benign and malignant lesions of the gingiva, thus posing difficulty in diagnosis. Excisional biopsy and histopathological findings help to confirm this lesion. Treatment consists of scaling, complete excision of the lesion, and extraction of teeth with poor prognosis. Though the recurrence rate for PCG of gingiva is less, long-term monitoring is recommended.
